# Imprecise Diagnosis of Diabetes Type in Youth: Prevalence, Characteristics, and Implications

**DOI:** 10.21203/rs.3.rs-2958200/v1

**Published:** 2023-05-25

**Authors:** Mustafa Tosur, Xiaofan Huang, Audrey S Inglis, Rebecca Schneider Aguirre, Maria J Redondo

**Affiliations:** Baylor College of Medicine, Texas Children’s Hospital; Baylor College of Medicine; Baylor College of Medicine; Baylor College of Medicine, Texas Children’s Hospital; Baylor College of Medicine, Texas Children’s Hospital

**Keywords:** pediatric diabetes, type 1 diabetes, type 2 diabetes, diabetes, misdiagnosis

## Abstract

Classifying diabetes at diagnosis is crucial for disease management but increasingly difficult due to overlaps in characteristics between the commonly encountered diabetes types. We evaluated the prevalence and characteristics of youth with diabetes type that was unknown at diagnosis or was revised over time. We studied 2073 youth with new-onset diabetes (median age [IQR]=11.4 [6.2] years; 50% male; 75% White, 21% Black, 4% other race; overall, 37% Hispanic) and compared youth with unknown versus known diabetes type, per pediatric endocrinologist diagnosis. In a longitudinal subcohort of patients with data for ≥3 years post-diabetes diagnosis (n=1019), we compared youth with unchanged versus changed diabetes classification. In the entire cohort, after adjustment for confounders, diabetes type was unknown in 62 youth (3%), associated with older age, negative IA-2 autoantibody, lower C-peptide, and no diabetic ketoacidosis (all, p<0.05). In the longitudinal subcohort, diabetes classification changed in 35 youth (3.4%); this was not statistically associated with any single characteristic. Having unknown or revised diabetes type was associated with less continuous glucose monitor use on follow-up (both, p<0.004). In sum, among racially/ethnically diverse youth with diabetes, 6.5% had imprecise diabetes classification at diagnosis. Further research is warranted to improve accurate diagnosis of pediatric diabetes type.

## Introduction

Pediatric diabetes is one of the most common chronic conditions in childhood. Type 1 diabetes (T1D) and type 2 diabetes (T2D) account for most pediatric diabetes cases, and their incidences increased significantly over the past decades [1]. T1D is characterized by absolute insulin deficiency, typically of autoimmune origin, requiring lifelong insulin treatment while T2D is marked by relative insulin deficiency with insulin resistance and, at least initially, may respond to lifestyle or non-insulin agents [2, 3]. However, overlaps in biological and clinical characteristics between different diabetes types pose significant challenges for clinicians [3]. Adding to the confusion, monogenic forms of diabetes, including neonatal and maturity-onset diabetes of the young (MODY), are relatively rare causes of pediatric-onset diabetes [4] that may resemble T1D or T2D but require genetic testing for diagnosis. Atypical forms of diabetes or atypical presentations of common forms of diabetes are more frequent in non-European individuals [5–9].

As each diabetes type requires different treatment approaches, determination of diabetes type at diagnosis of diabetes guides clinical management decisions in both short- and long-term diabetes care [3]. It also shapes the type and depth of diabetes education and counseling that families receive during their clinical encounters. In addition, clinicians follow screening recommendations for commonly associated conditions (e.g., other autoimmune diseases) and micro- and macrovascular complications of diabetes based on the patient’s assigned diabetes type. Finally, diabetes risk in family members and approaches to prevention vary by diabetes type. Therefore, precise determination of diabetes type at the onset of diabetes is key to providing optimal diabetes care.

Although there is heightened interest in the challenges surrounding diagnosis of diabetes type [5, 10–18], the prevalence of imprecise diagnosis of pediatric diabetes types and the factors associated with imprecise diagnosis are not fully understood. Therefore, we aimed to study imprecise diagnosis of diabetes type in racially and ethnically diverse youth. We hypothesized that a sizeable percentage of children with diabetes have an undetermined type of diabetes at diagnosis of diabetes and/or their assigned diabetes types change over time. Better understanding the frequency and characteristics of children with imprecise diagnosis of diabetes type at onset will facilitate targeted interventions to address this problem.

## Methods

### Participants

**Figure 1:**
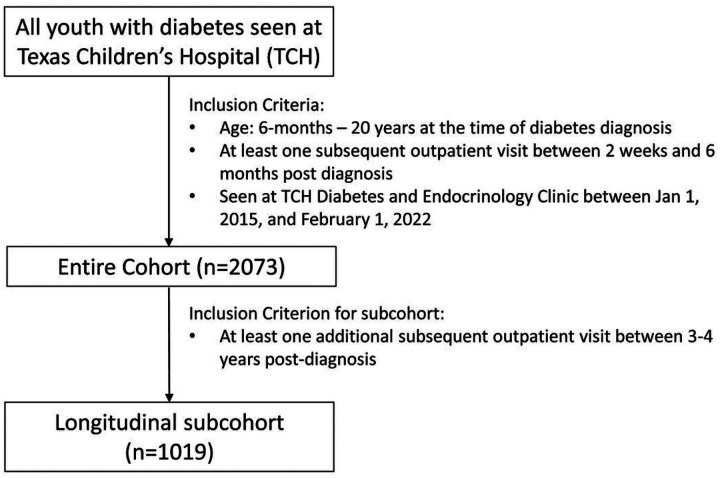
Flow diagram of study participants

In this retrospective study, we included individuals with any type of diabetes who were between six months and 20 years old at the time of diabetes diagnosis, had at least one subsequent outpatient visit between 2 weeks and 6 months post-diagnosis, and were seen at a Texas Children’s Hospital (TCH) Diabetes and Endocrinology Clinic between January 1, 2015, and February 1, 2022 (n = 2073, entire cohort). In addition, we studied a subcohort of patients who had at least an additional outpatient visit between 3 and 4 years post-diagnosis (n = 1019, longitudinal subcohort) (Fig. 1). The study was approved by the Baylor College of Medicine Institutional Review Board (IRB) (H-47418), and the requirement for informed consent was waived. All research was performed according to relevant guidelines and regulations.

### Procedures

With the assistance of a TCH electronic medical record (EMR) (i.e., Epic) data specialist, we generated a subject list meeting above inclusion and exclusion criteria, and extracted the following variables from the office visits that occurred after the diagnosis of diabetes and 3–4 years post-diagnosis: age, sex, race, ethnicity, body mass index (BMI) percentile, C-peptide, glucose, hemoglobin A1c (HbA1c), presence of diabetic ketoacidosis (DKA), and the results for autoantibodies (Glutamic Acid Decarboxylase [GAD], Islet Antigen 2 [IA-2], Anti-insulin [IAA], and Zinc Transporter 8 [ZnT8]). The race and ethnicity categorizations were based on self-report per documentation in the electronic medical record. We used the following racial and ethnic categories: (1) Races: White, African American, and Other races; and (2) Ethnicities: Hispanic and non-Hispanic.

Per clinical protocol at TCH, all pediatric endocrinologists must document diabetes type in a designated section (“flowsheet”) of the EMR at each diabetes encounter, by selecting one of the options including T1D, T2D, steroid-induced diabetes, cystic fibrosis-related diabetes, gestational diabetes, MODY, neonatal diabetes, and unknown diabetes type. Per clinical protocol, all patients with new-onset diabetes have islet autoantibodies, random C-peptide and random glucose tested at the time of diagnosis. This information is available for the treating pediatric endocrinologist at time of the first outpatient visit, along with other clinical and demographic characteristics. Diabetes type at first outpatient visit and at an outpatient visit that occurred between 3–4 years post-diagnosis were also extracted and were classified as one of the following: type 1, type 2, other (MODY steroid-induced, cystic fibrosis [CF]-related, other-not specified), and unknown.

### Statistical Analyses

Patient characteristics were summarized by median with 25th and 75th percentile, mean with standard deviation, or frequency with proportion. Descriptive statistics were stratified by whether the type of diabetes was known or unknown at the first office visit after diabetes diagnosis, and changed or unchanged three years post-diagnosis. The characteristics of the study population were compared using the Wilcoxon Rank Sum test or Pearson Chi-square test. Univariable logistic regression was used to identify baseline characteristics that were significantly associated with unknown or changed diabetes type. All the significant factors from univariable model were included in multiple logistic regression model to analyze if unknown or changed diabetes type was significantly associated with HbA1c at 6–12 months after diagnosis after adjusting for age, gender, race, ethnicity, HbA1c at diagnosis, and duration from diabetes diagnosis to the HbA1c at 6–12 months. A significance level of 0.05 was used.

### Definitions

Unknown diabetes type: Diabetes of unknown type as documented by the treating pediatric endocrinologist in the EMR flowsheet section at each outpatient diabetes encounter.

#### Changed diabetes type:

**Figure 2:**
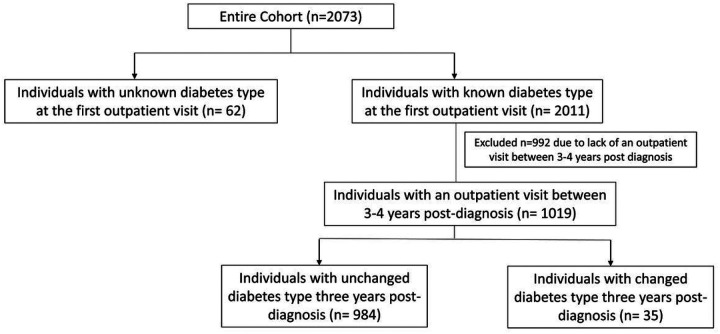
Study design

Diabetes type that was different at the time of the first outpatient visit and an outpatient visit between 3–4 years after diagnosis, both as documented by the treating pediatric endocrinologist in the EMR flowsheet. In the analysis, the diagnosis type at three years post-diagnosis was considered the correct diagnosis type. Patients initially diagnosed with an unknown diabetes type who were then reclassified to a known diabetes type were not included in the count of unchanged diabetes type (Fig. 2).

## Results

We studied 2073 children with new onset diabetes who met our inclusion criteria (Entire Cohort). The median age [IQR] at diagnosis was 11.4 [6.2] years and 50% were male. Racial distribution was 75% White, 21% Black and 4% other races. Overall, 37% were Hispanic. The median HbA1c [IQR] at diagnosis was 11.4% [3.4] and median BMI [IQR] was 85 [39] percentile. Twenty-six percent presented with DKA at diagnosis. The most common diabetes types were T1D (73%) followed by T2D (21%) while diabetes type was unknown in 3% (n = 62) of the cohort at diagnosis of diabetes. Baseline characteristics are summarized in Table 1.

Compared to those with known diabetes type at diagnosis, children with unknown diabetes type were older (p < 0.001), less likely to be positive for GAD-65 or IA-2 autoantibodies (both p < 0.001), and had higher median BMI percentile (p < 0.001) and random C-peptide level (p < 0.001) (Table 1). They also had significantly lower percentage of DKA at diagnosis (p < 0.001). Use of continuous glucose monitoring device was lower at initial office visit after diabetes diagnosis (p = 0.01) and remained persistently lower three years post-diagnosis in this group than among children with known diabetes type (p = 0.003). In a multivariable linear regression analysis including age, sex, race, ethnicity, BMI percentile, C-peptide, presence of DKA, GAD antibody positivity, IA-2 antibody positivity, unknown diabetes type at diagnosis was associated with older age (p = 0.047), negative IA-2 autoantibody (p = 0.005), lower C-peptide (p = 0.004), and absence of DKA (p = 0.024) after adjustment for confounding factors (Table 2).

Of 2073 children, 1019 (49%) of them had follow up data available between 3 and 4 years after diabetes diagnosis (longitudinal subcohort) (Table 3). In this subcohort, 35 patients (3.4%) were identified as having a revised diagnosis of diabetes type three years after onset while diabetes classification remained unchanged in 984 patients (96.6%). Of these 35 patients with a revised diabetes classification, approximately half them were initially diagnosed as having T1D (49%, n = 17), five (14%) were diagnosed with T2D, and 13 (37%) were diagnosed with other diabetes types (Table 3). After three years, most of the patients with changed diabetes type were diagnosed with T2D (66%, n = 23), six patients were diagnosed with T1D (17%), three (9%) patients with other diabetes types, and three patients (9%) with unknown diabetes type (Table 3). As indicated in Methods, children with unknown diabetes type at diagnosis were not included in the category of children with changed diabetes type even if their diabetes was reclassified to a known type at the three-year follow-up visit.

Compared to those with unchanged diabetes type three years post-diagnosis, children with changed diabetes type were older (p < 0.001), more likely to be of Hispanic ethnicity (p < 0.001) and negative for autoantibodies to GAD65 (p < 0.001) and IA-2 (p < 0.001), and had higher C-peptide (p < 0.001) but lower glucose (p = 0.01) and HbA1c (p = 0.01) levels at diagnosis (Table 3). They also had significantly lower percentage of DKA at diagnosis (p = 0.01). Use of continuous glucose monitoring device was lower at initial office visit after diabetes diagnosis (p = 0.03) and remained persistently lower three years post-diagnosis (p < 0.001) in this group than children with unchanged diabetes type. In a multivariable linear regression analysis including age, sex, race, ethnicity, BMI percentile, C-peptide, presence of DKA, GAD antibody positivity, IA-2 antibody positivity, HbA1c, and glucose, there was no individual variable associated with changed diabetes type after adjustment for confounding factors (data not shown).

## Discussion

The prompt and accurate diagnosis of diabetes type is an ongoing issue in the pediatric population. We found that, in a racially and ethnically diverse cohort, the type of diabetes is unknown at the time of diabetes diagnosis or changes three years later in 6.5% of youth (one in 15 children) with diabetes mellitus. Children with unknown or changed diabetes type were less likely to use a continuous glucose monitor than children with timely diabetes classification. Imprecise diagnosis may lead to suboptimal diabetes education, treatment and screening for associated conditions and complications and therefore, carry higher risk of negative health outcomes. Additionally, not receiving a diagnosis of diabetes type or having this revised over time can harm the trust built between families and their diabetes care providers. Therefore, accurately and timely diagnosis of diabetes type at the time of diabetes onset is crucial..

As expected, T1D was the most common diabetes type in our cohort, both at diagnosis and three years later. However, other diabetes types were more common three years after onset among children who at onset received an imprecise diagnosis of diabetes type, that is, who were classified as having unknown type of diabetes or whose classification was revised. This finding suggests a delay in diagnosing pediatric diabetes types other than T1D, such as T2D or MODY. At onset, children were over diagnosed as having T1D while other diabetes types were underdiagnosed. Hispanic ethnicity was significantly more frequent among the children with changed diabetes type (66%) compared with those with stable classification (33%), possibly due to the difficulties to accurately diagnose pediatric T2D at onset and the increasing rates of T2D disproportionately affecting Hispanic and Black youth [1].

The misdiagnosis of T1D in a patient with T2D could result in unnecessary insulin treatment when diet and lifestyle modifications along with non-insulin medications may be more appropriate [19]. Insulin treatment increases the risk of hypoglycemia [20], which is associated with adverse effects on the central nervous system and higher risk of cardiovascular events and mortality, especially during a severe episode [21]. In addition, insulin treatment is associated with significant emotional distress in individuals with diabetes and their families [22, 23]. On the contrary, initial misclassification of T1D as another type of diabetes or lack of a timely diagnosis of T1D (i.e., unknown diabetes type) may place children at greater risk of DKA, a life-threatening complication of diabetes [24–26]. DKA is associated with several short- and long-term adverse health outcomes such as decline in memory and intelligence quotient [27], altered brain structure [28], kidney injury [29], cerebral venous thrombosis [30], decreased likelihood of having partial remission [31] and negative impact on glycemic control over time [32, 33]. Therefore, establishing a precise diagnosis of diabetes type in a timely manner is of paramount importance for optimal health outcomes.

In the present study, older age, negative IA-2 antibody, lower C-peptide, and absence of DKA at the onset of diabetes emerged as significant factors associated with unknown diabetes type limiting clinicians’ ability to determine diabetes type at the time of diagnosis of diabetes. Considering the biochemical and clinical characteristics of T1D and T2D [3, 34], this observation suggests that a lack of a typical characteristics of T1D at diagnosis of diabetes may lead to confusion in determination of diabetes type by clinicians. Furthermore, C-peptide levels measured at diagnosis are difficult to interpret due to possible transient beta-cell function suppression due to extreme hyperglycemia, ketosis and/or DKA. A substantial percentage of those with “unknown diabetes type” at the onset of diabetes were diagnosed with T2D at 3–4 years post-diagnosis. This aligns with our observation that a lack of T1D-suggestive phenotypic features in children plays an important role in delayed determination of diabetes type and underscores the urgent need for better diagnostic tools and biomarkers to confirm the timely diagnosis of T2D in children. Dramatic increase in the incidence of pediatric T2D in recent decades [1], more rapid beta-cell functional deterioration [35] and earlier onset of diabetes complications [36–38] in youth with T2D compared with their adult counterparts only augment the gravity of this public health problem and call all stakeholders to take immediate actions.

Delayed diagnosis or misdiagnosis of diabetes type at diagnosis of diabetes in children is due, in part, to limitations of the current classification system of diabetes [2], which offers clinicians only limited options of diabetes types despite tremendous heterogeneity of diabetes [39]. Although distinct categorization of diabetes types seems clinically more practical in real-word settings, evidences supporting the complexity of diabetes and the existence of a spectrum of diabetes phenotypes with differential contributions of multiple pathophysiologic processes in concert with each other as suggested by Palette Model are accumulating [3, 40]. This led to multiple efforts by us and other investigators looking into improved classification of diabetes types and subtypes to allow for personalized treatment approaches [5, 10, 16, 41, 42].

The limitations of our study include its retrospective design and having three-year follow-up data for only half of the cohort. The study strengths include a large, racially/ethnically diverse pediatric patient population and availability of widely used biological and clinical data that allowed us to examine clinically impactful factors in the context of our research question.

In conclusion, one in 15 children is affected by imprecise diagnosis of diabetes type in a racially and ethnically diverse pediatric diabetes population. Multifaceted effects of imprecise diagnosis of diabetes type in health outcomes warrant further research to better understand the underlying root causes and offer impactful solutions. These efforts may include improved classification of diabetes and the incorporation in clinical practice of biomarkers (e.g., genetics) to characterize diabetes in children.

## Figures and Tables

**Table 1:** Demographics and clinical characteristics of patients with “unknown” diabetes type vs. known diabetes type.

Demographic or clinical characteristic	N	Overall cohort	Known diabetes type (n = 2011)	Unknown diabetes type (n = 62)	p-value
**Age**, years	2073	11.4 (6.2)	11.3 (6.2)	13.6 (3.5)	**<0.001**
**Male**, n (%)	2073	1028 (50%)	1002 (50%)	26 (42%)	0.22
**Race**, n (%)	2023				0.34
White		1516 (75%)	1475 (75%)	41 (67%)	
Black or African American		417 (21%)	400 (20%)	17 (28%)	
Other		90 (4%)	87 (4%)	3 (5%)	
**Ethnicity**, n (%)	2042				0.06
Hispanic or Latino		756 (37%)	726 (37%)	30 (48%)	
Not Hispanic or Latino		1286 (63%)	1254 (63%)	32 (52%)	
**BMI percentile**	1562	85 (39)	84 (40)	97 (9)	**<0.001**
**GAD autoantibody positive**, n (%)	2073	1606 (77%)	1571 (78%)	35 (56%)	**<0.001**
**IA-2 antibody positive**, n (%)	2073	1559 (75%)	1525 (76%)	34 (55%)	**<0.001**
**Insulin autoantibody positive**, n (%)	2073	1129 (54%)	1097 (55%)	32 (52%)	0.65
**C-peptide**, ng/mL	1407	0.54 (0.79)	0.55 (0.77)	1.36 (1.10)	**<0.001**
**Glucose**, mg/dL	1188	312 (216)	313 (216)	280 (170)	0.56
**HbA1c**, %	1519	11.4 (3.4)	11.4 (3.4)	12.0 (3.5)	0.26
**Presence of DKA**, n (%)	2073	548 (26%)	544 (27%)	4 (6%)	**<0.001**
**Use of CGM at first visit**, n (%)	1925	336 (17%)	334 (18%)	2 (4%)	**0.01**
**Use of CGM three years post-diagnosis** n (%)	1007	484 (48%)	479 (49%)	5 (19%)	**0.003**
**Diabetes type at initial diagnosis**, n (%)	2073				**<0.001**
Type 1		1519 (73%)	1519 (76%)	0 (0%)	
Type 2		440 (21%)	440 (21%)	0 (0%)	
Other		52 (3%)	52 (3%)	0 (0%)	
Unknown		62 (3%)	0 (0%)	62 (100%)	
**Diabetes type three years post-diagnosis**, n (%)	1019				**<0.001**
Type 1		831 (82%)	820 (83%)	11 (41%)	
Type 2		163 (16%)	154 (16%)	9 (33%)	
Other		18 (2%)	15 (2%)	3 (12%)	
Unknown		7 (1%)	3 (0%)	4 (15%)	

Unless otherwise specified, all continuous variables were expressed as median (IQR). Wilcoxon rank sum test and Chi-square test were used as appropriate. N is the number of non-missing values.

**Table 2:** Association of baseline characteristics with unknown diabetes types in a multivariable linear regression analysis including age, sex, race, ethnicity, BMI percentile, C-peptide, presence of DKA, GAD65 antibody positivity and IA-2 antibody positivity.

Variable	OR	95% CI	p-value
Age	1.15	1.01–1.32	**0.047**
Sex	1.04	0.46–2.36	0.929
Black or African American Race	2.44	0.68–9.52	0.180
Other race	2.50	0.12–17.75	0.426
Not Hispanic or Latino Ethnicity	0.41	0.11–1.38	0.163
BMI percentile	1.01	0.99–1.04	0.260
GAD autoantibody positivity	0.34	0.10–1.05	0.066
IA-2 antibody positivity	0.17	0.04–0.56	**0.005**
C-peptide	0.54	0.35–0.78	**0.004**
Presence of DKA	0.23	0.05–0.72	**0.024**

**Table 3:** Demographics and clinical characteristics of patients with “changed” diabetes type from diagnosis to three years post-diagnosis vs. unchanged diabetes type.

Demographic or clinical characteristic	N	Overall cohort	Unchanged diabetes type (n = 984)	Changed diabetes type (n = 35)	p-value
**Age**, years	1019	10.7 (5.7)	10.5 (5.9)	13.6 (3.6)	**<0.001**
**Male**, n (%)	1019	515 (51%)	496 (50%)	19 (54%)	0.65
**Race**, n (%)	1002				0.16
White		772 (77%)	748 (77%)	24 (71%)	
Black or African American		190 (19%)	180 (19%)	10 (29%)	
Other		40 (4%)	40 (4%)	0 (0%)	
**Ethnicity**, n (%)	1002				**<0.001**
Hispanic or Latino		337 (34%)	314 (32%)	23 (66%)	
Not Hispanic or Latino		665 (66%)	653 (68%)	12 (34%)	
**BMI percentile**	670	82.7 (41.5)	81.0 (41.3)	98.2 (3.9)	**<0.001**
**GAD autoantibody positive**, n (%)	1019	807 (79%)	790 (80%)	17 (49%)	**<0.001**
**IA-2 antibody positive**, n (%)	1019	788 (77%)	773 (79%)	15 (43%)	**<0.001**
**Insulin autoantibody positive**, n (%)	1019	565 (55%)	550 (56%)	15 (43%)	0.13
**C-peptide**, ng/mL	719	0.47 (0.66)	0.46 (0.60)	1.79 (1.05)	**<0.001**
**Glucose**, mg/dL	608	316 (226)	321 (226)	246 (169)	**0.01**
**HbA1c**, %	747	11.4 (3.3)	11.5 (3.3)	10.3 (4.4)	**0.01**
**Presence of DKA**, n (%)	1019	272 (27%)	269 (27%)	3 (9%)	**0.01**
**Use of CGM at first visit**, n (%)	930	125 (13%)	125 (14%)	0 (0%)	**0.03**
**Use of CGM three years post-diagnosis** n (%)	1007	484 (48%)	482 (50%)	2 (6%)	**<0.001**
**Diabetes type at initial diagnosis**, n (%)	1019				**<0.001**
Type 1		831 (82%)	814 (83%)	17 (49%)	
Type 2		136 (13%)	131 (13%)	5 (14%)	
Other		25 (1%)	12 (1%)	13 (37%)	
Unknown		27 (3%)	27 (3%)	0 (0%)	
**Diabetes type three years post-diagnosis**, n (%)	1019				**<0.001**
Type 1		831 (82%)	825 (84%)	6 (17%)	
Type 2		163 (16%)	140 (14%)	23 (66%)	
Other		18 (2%)	15 (3%)	3 (9%)	
Unknown		7 (1%)	4 (0%)	3 (9%)	

Unless otherwise specified, all continuous variables were expressed as median (IQR). Wilcoxon rank sum test and Chi-square test were used as appropriate. N is the number of non-missing values.

## Data Availability

The datasets generated during and/or analyzed during the current study are available from the corresponding author on reasonable request.
